# Optimizing care in bipolar disorder: focus on prevention and early intervention

**DOI:** 10.1192/j.eurpsy.2026.10411

**Published:** 2026-07-31

**Authors:** L. Yatham

**Affiliations:** University of British Columbia, Vancouver, Canada

## Abstract

**Abstract:**

Despite treatment in health care settings, patients with bipolar disorder are well only half of the time; the other 50% of the time is spent experiencing syndromal or sub-syndromal mood symptoms which in addition to increasing the risk of suicide can also significantly impact functioning and quality of life. This presentation will review opportunities and challenges for intervening early during clinical high risk stages and in the early stages of bipolar disorder and examine if such strategies can improve outcomes.

Several challenges exist for intervening during “high risk” stages. First, high risk stages for BD can be defined only clinically at present, and no biomarkers exist that can increase the prediction of conversion to BD. Indeed, current data suggest that only upto 20% of patients defined as high risk for BD convert to BD I or BD II. Further, most studies that assessed the efficacy of interventions in clinically high risk BD to date focussed on the impact of interventions mostly on improving sub-syndromal symptoms. However, a small RCT that assessed the efficacy of multifamily focussed therapy reported lower rates of conversion to BD compared with the wait list control offering some promise for intervention strategies in high risk stages to improve outcomes. Since BD onset is often associated with increase in inflammation and oxidative stress markers, strategies targeting these areas might be beneficial in preventing or delaying the onset of BD.

A firm diagnosis of BD can only be made after a patient has had their first manic or hypomanic episode. Optimizing treatment at the first manic episode may also improve outcomes. This presentation will review outcome data from a first episode mania cohort and present results of clinical, cognitive and functional outcomes in first episode cohorts.

**Disclosure of Interest:**

L. Yatham Grant / Research support from: Received grant support from Sumitomo and Abbvie in the last 3 years, Consultant of: Sumitomo Pharam, Alkermes, LivaNova, Puretech, Neurotorium,

